# Gastric Metastases From Invasive Breast Lobular Carcinoma, Identified by [ ^18^F]FDG PET/CT, 20 Years After Primary Diagnosis: A Case Report

**DOI:** 10.1002/ccr3.70881

**Published:** 2025-09-09

**Authors:** Alina Diana Ilonca, Amélie Gudin‐de‐Vallerin, Sophie Guillemard, Marie‐Claude Eberle, Jeanne Briant, Séverine Guiu, Emmanuel Deshayes, Cyril Fersing

**Affiliations:** ^1^ Nuclear Medicine Department, Institut Régional du Cancer de Montpellier (ICM) University Montpellier Montpellier France; ^2^ Pathology Department, Institut Régional du Cancer de Montpellier (ICM) University Montpellier Montpellier France; ^3^ Radiotherapy Department, Institut Régional du Cancer de Montpellier (ICM) University Montpellier Montpellier France; ^4^ Medical Oncology Department, Institut Régional du Cancer de Montpellier (ICM) University Montpellier Montpellier France; ^5^ Institut de Recherche en Cancérologie de Montpellier (IRCM) INSERM U1194, University Montpellier, Institut régional du Cancer de Montpellier (ICM) Montpellier France; ^6^ IBMM, Univ Montpellier, CNRS, ENSCM Montpellier France

**Keywords:** case report, gastrointestinal metastases, immunohistochemistry, lobular carcinoma, PET/CT imaging

## Abstract

Invasive lobular carcinoma (ILC) of the breast is a rare subtype of breast cancer with distinct metastatic patterns. Although gastrointestinal metastases are rare, they can occur years after initial treatment. This case highlights the diagnostic challenges and management of late‐onset gastric metastases. A 68‐year‐old woman with a history of ILC treated 20 years earlier presented with elevated tumor markers. [^18^F]fluorodeoxyglucose positron emission tomography/computed tomography (FDG PET/CT) revealed hypermetabolic lesions in the stomach and esophagus in this patient with previously diagnosed gastritis and gastroesophageal reflux disease. Endoscopy and biopsies confirmed the presence of metastatic ILC in the stomach. Adjustment of treatment, including exemestane and everolimus, followed by paclitaxel and tamoxifen, resulted in partial disease control. Late‐onset gastrointestinal metastases of ILC are uncommon and require special vigilance, particularly in patients with associated benign gastrointestinal pathologies, which may delay diagnosis. Persistent or new‐onset gastrointestinal symptoms in breast cancer patients warrant thorough evaluation, including FDG PET/CT imaging and histological confirmation.

## Graphical abstract

FDG PET/CT demonstrating a focal area of increased metabolic activity in the gastric region (blue arrows; SUVmax 6.3), shown on maximum‐intensity projection (MIP) images (A), axial PET slice (B), and axial fused PET/CT slices (C, D). This finding corresponds to gastric metastases from invasive lobular breast carcinoma in a patient treated 20 years earlier.

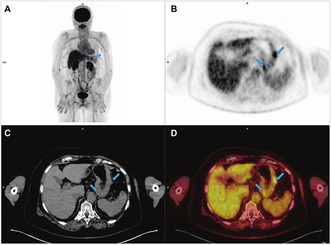

**Summary**

- Focal stomach uptake on [^18^F]FDG PET/CT in patients with a history of breast cancer should raise suspicion of late‐onset metastatic lesions, even many years after diagnosis and in the presence of gastritis.

## 1 Introduction

Breast cancer remains the most common cancer and one of the leading causes of death in women [1, 2]. Invasive lobular carcinoma (ILC) represents approximately 5%–15% of invasive breast carcinomas, with an incidence between 1% and 20% in the literature, increasing in the last 20 years, possibly related to hormone replacement therapy, compared to ductal carcinoma, which remains stable [3]. At diagnosis, from 3% to 12% of patients are metastatic, and 30%–80% of patients experience distant metastatic progression after surgery, chemotherapy, hormone therapy, and radiotherapy [4, 5]. The preferred metastatic sites are the lymph nodes, skeleton, gynecological organs, and peritoneum [6]. Extrahepatic digestive metastases remain rare, with incidences reported in the literature between 6% and 18%, sometimes synchronous with the diagnosis of the primary lesion [7], and sometimes appearing years after the diagnosis of the breast lesion [8, 9, 10]. The stomach is more frequently affected compared to the colon (6%–18% vs. 8%–12%) [11].

We present the case of a female patient with metastatic infiltrating lobular carcinoma, who developed unexpected secondary gastric metastases 20 years after the management of the primary tumor. To the best of our knowledge, this is the first report on the identification of such lesions by [^18^F]fluorodeoxyglucose (FDG) positron emission tomography/computed tomography (PET/CT). Other metastatic sites, particularly bone, were controlled under treatment. Notably, the patient had a history of gastroesophageal reflux and histologically confirmed *Helicobacter pylori* ‐negative gastritis, which might have misled the diagnosis toward a benign condition.

## 2 Case Description

**FIGURE 1:**
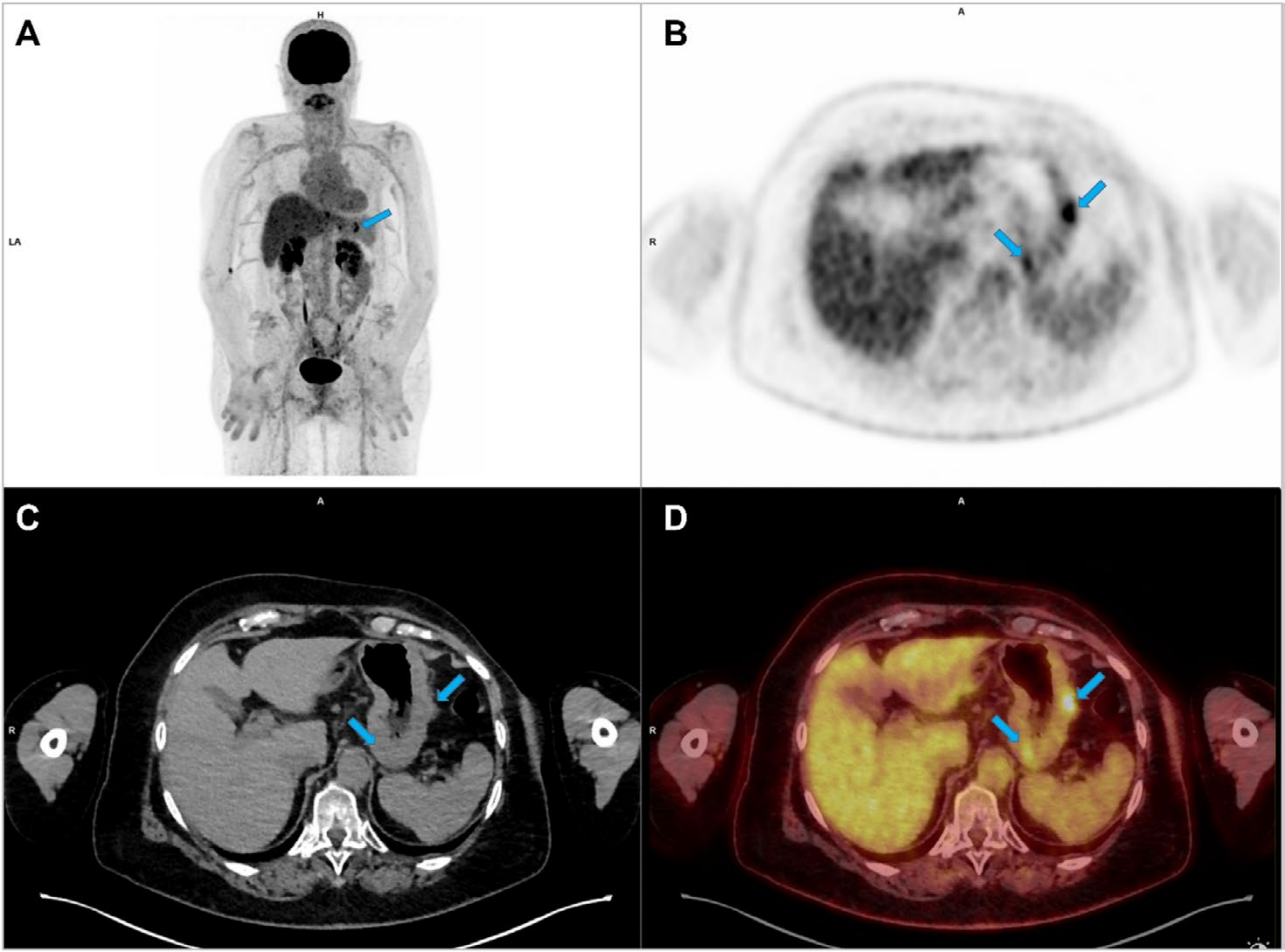
FDG PET/CT imaging showing a focal and high metabolism areas in the gastric region (blue arrows), SUVmax 6.3, on MIP (maximum‐intensity projection) images (A), axial PET slice (B) and axial fused PET/CT slice (C, D).

The patient is a 68‐year‐old woman treated in 2003 for ILC of the left breast with mastectomy and axillary dissection, stage pT3N1 (estrogen receptors 90%, progesterone receptors 100%, HER2 negative, Ki‐67 5%). She received 6 cycles of FAC 50 chemotherapy (5‐fluorouracil, adriamycin, cyclophosphamide), radiotherapy, and anastrozole for 6 years. In June 2015, she had elevated tumor markers and several secondary bone lesions diagnosed on FDG PET/CT. Thus, treatment with letrozole and denosumab began in August 2015, along with radiotherapy for the T7–T8 vertebrae due to minimal epidural involvement. Denosumab was stopped in 2017 due to dental care. New bone progression in December 2019 led to treatment with fulvestrant and abemaciclib at decreasing doses (to balance efficacy and tolerance), achieving good control of bone metastatic disease, with complete metabolic response on several follow‐up FDG PET/CT. In June 2023, slightly increased carcinoembryonic antigen (CEA) (from 59 to 62 μg/L; normal values < 3.0 ng/mL) and cancer antigen 15‐3 (CA15‐3) (from 24 to 31 IU/mL; normal values < 30 IU/mL) levels were observed without signs of progression on FDG PET/CT. By October 2023, further increases in CEA (from 62 to 97 μg/L) and CA15‐3 (from 31 to 36 IU/mL) led to a new FDG PET/CT, which showed three new focal uptakes of FDG: one in the cardia region (Standardized Uptake Value (SUV) max 6.9), and two in the gastric areas of the fundus and greater curvature (SUV max 6.3) (Figure 1). The patient had been on topical gastric protectors since June 2021 and esomeprazole since April 2022 for gastroesophageal reflux and *Helicobacter pylori* ‐negative gastritis, histologically proven in 2019.

## 3 Diagnostic Assessment

### 3.1 Endoscopic Examination

**FIGURE 2:**
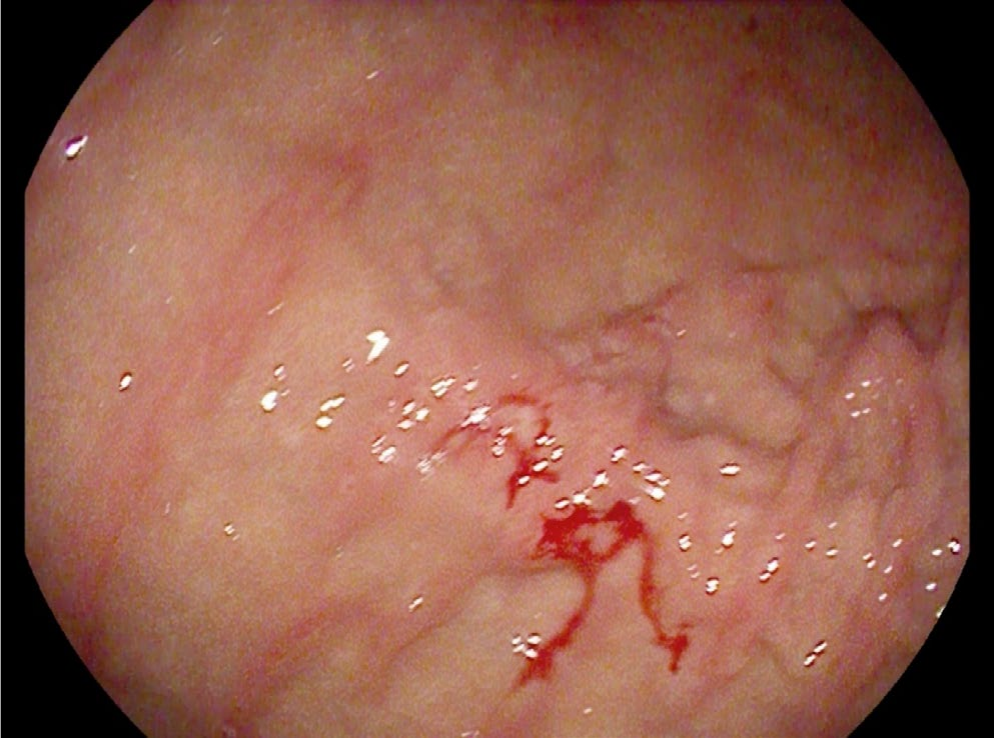
Endoscopy images showing ulcerations of the fundic region.

Regarding the results of the FDG PET/CT, an endoscopic examination revealed two ulcerated lesions of 3 cm and 2 cm at the antral‐fundic junction, which were indurated, and one linear ulceration under the cardia, associated with diffuse gastritis (Figure 2). Biopsies were taken from these lesions for further exploration. The colonoscopy was normal, except for internal hemorrhoids.

### 3.2 Histopathological Examination

**FIGURE 3:**
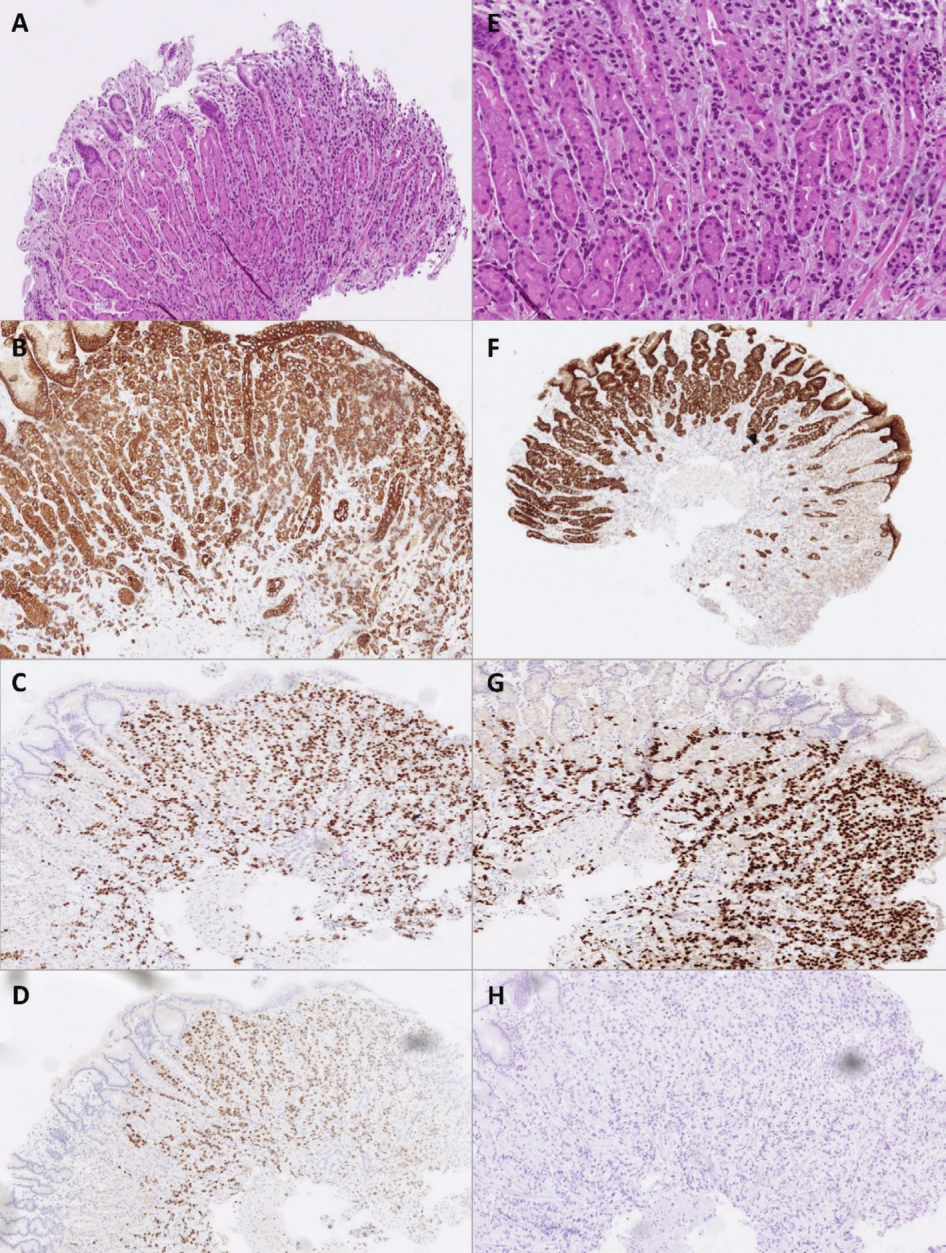
Representative images of immunohistochemistry staining results showing hematoxylin–eosin‐saffron (HES) staining (A, E), CKAE1/AE3 (B), E‐cadherin (F), GATA3 (C), TRPS1 (G), estrogen receptor (D) and progesterone receptor (H).

The histopathologic analysis of the gastric biopsy, corresponding to three ulcerated lesions, highlighted a carcinomatous proliferation, with morphology (proliferation of small cells that lack cohesion, arranged in single‐file linear cords, having round or notched ovoid nuclei and a thin rim of cytoplasm, with occasional intracytoplasmic lumen) and immunophenotype (GATA3+/TRPS1+/Cdx2−/E‐Cadherin‐) indicative of secondary localizations of lobular carcinoma on a background of *H. pylori* ‐negative antral gastritis. Immunohistochemical analysis confirmed the expression of estrogen receptors at 80%, the absence of significant progesterone receptor expression, and no HER2 expression with a 1+ score (Figure 3). The Ki‐67 proliferation index was estimated at 6%. The heterogeneity of hormone receptor or HER2 expression in metastatic disease, compared to the primary lesion, including the loss or gain of receptor expression, is a well‐known phenomenon in breast cancer [12].

## 4 Outcomes

**FIGURE 4:**
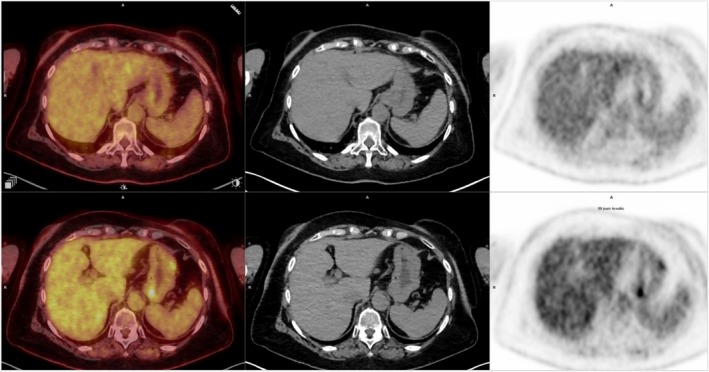
Comparison of FDG PET/CT images taken in April 2024 (top) and October 2023 (bottom), on axial fused PET/CT slice (left, middle) and axial PET slice (right), showing a partial response of the cardia lesion and a complete responses of other gastric lesions, with disappearance of the focal uptake in the antral‐fundic region.

The diagnosis of gastric metastases had a significant impact on management, and a new therapeutic strategy with exemestane 25 mg/day in combination with everolimus at a progressive dose was introduced in January 2024. There was a partial metabolic response of the cardia lesion and a complete metabolic response of other gastric lesions on the FDG PET/CT performed on April 2024 (Figure 4).

**FIGURE 5:**
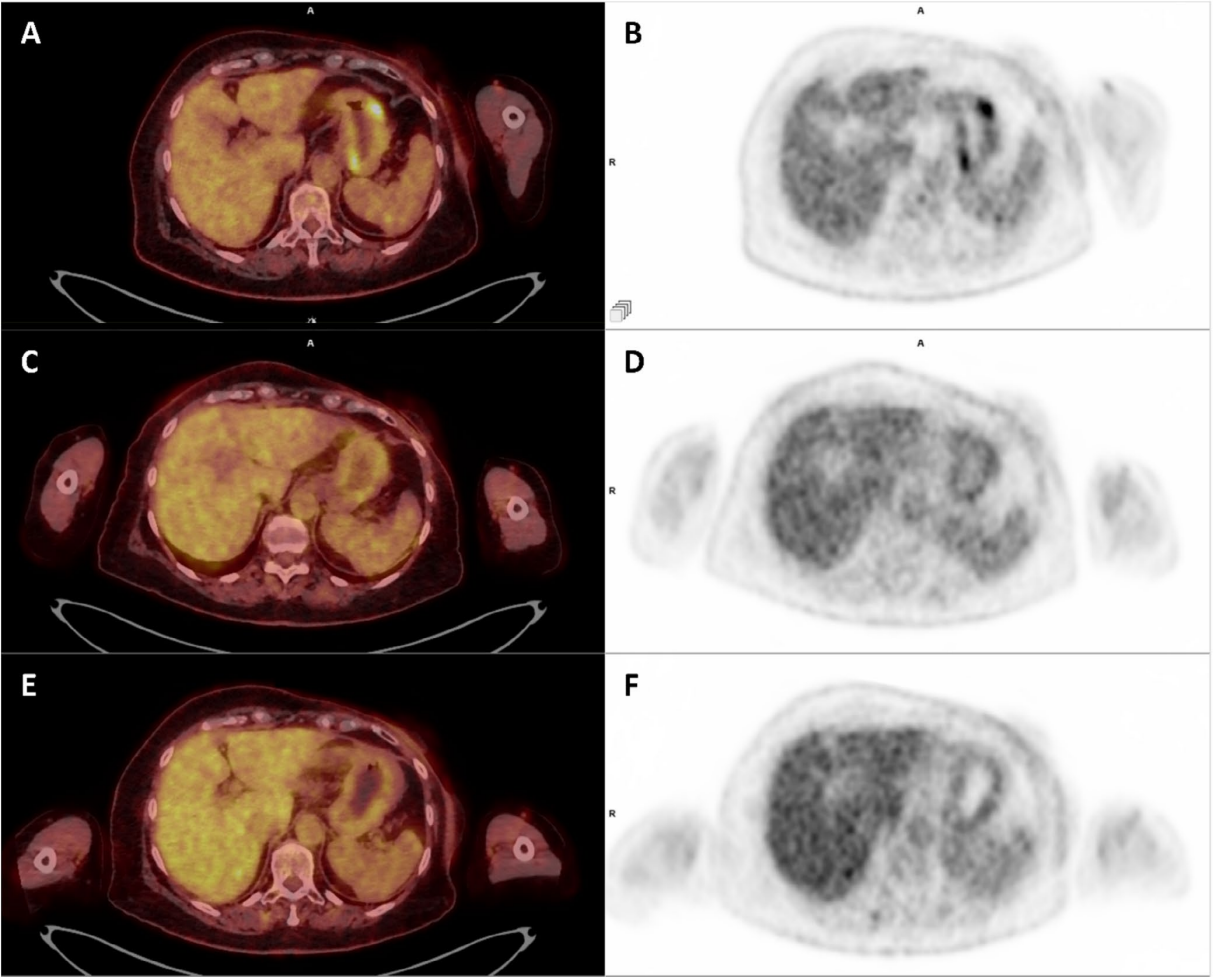
Comparison of FDG PET/CT images taken in July 2024 (A, B), October 2024 (C, D) and January 2025 (E, F), on axial fused PET/CT slice (A, C, E) and axial PET slice (B, D, F).

However, the patient developed grade 2 interstitial pneumonitis under everolimus, which was stopped in April 2024, followed by a progressive resumption of gastric lesions on the FDG PET/CT of July 2024. A new line of treatment with weekly paclitaxel was started in July 2024, with a partial metabolic response of gastric lesions, stopped in October 2024 due to peripheral neuropathy. Following this, treatment with tamoxifen was initiated and was clinically well tolerated, with therapy still ongoing. Reassessment with FDG PET/CT in January 2025, 2 months after starting treatment, demonstrated relative stability in terms of uptake intensity and volume of the metastatic gastric lesions (Figure 5).

## 5 Discussion and Teaching Points

The diagnosis of gastrointestinal metastases from breast cancer is not always obvious and remains a fairly rare occurrence. Clinical symptoms are nonspecific, such as epigastric pain, nausea, vomiting, dyspepsia, and dysphagia [13], or they may be absent, especially in nonobstructive forms. These symptoms may be attributed to pre‐existing digestive pathologies, as in the case of our patient, who had been treated for years for gastritis and gastroesophageal reflux. This can delay the diagnosis, making it more difficult in patients with associated digestive pathologies.

Digestive metastases as the first distant metastatic site are rare in invasive lobular breast cancer [14, 15]. They typically appear a few years after the primary breast lesion diagnosis, with a peak at 2 years for patients who received only local treatment for estrogen receptor‐negative tumors [16]. However, they can appear much later, with delays of 6–30 years reported for other histological forms [17, 18].

In the patient described in this report, gastric metastases appeared exceptionally late, 20 years after treatment of the primary lesion, while secondary bone metastases were in complete response to treatment for 4 years. The warning sign was a progressive increase in tumor markers, despite no signs of progression on an initial FDG PET/CT. The follow‐up FDG‐PET scan performed 3 months later revealed a moderate‐intensity uptake pattern with hypermetabolism in the stomach. The focal nature of the uptake led to further endoscopic exploration in this patient, who had been followed for years for histologically proven gastritis. While this finding could have been associated with the patient's long‐standing, histologically proven gastritis, it ultimately led to an endoscopic evaluation, which confirmed secondary digestive localizations of her breast carcinoma.

Lobular carcinomas are likely to show low FDG avidity; therefore, even a moderate increase in digestive hypermetabolism, especially when focal uptakes, warrants further investigation and remains challenging to assess using FDG PET/CT imaging alone [19]. Even in the absence of gastritis or gastric tumors, analysis of FDG uptake in the stomach remains difficult due to physiological uptake that may be high. In such cases of tumors presenting with overexpression of estrogen receptors, a [^18^F]fluoroestradiol PET/CT examination could have facilitated the diagnosis or enabled earlier identification of progressive lesions. Additionally, emerging FAPI PET tracers, targeting cancer‐associated fibroblasts, hold promise for this indication and should be thoroughly evaluated to determine their potential clinical utility in this context [20, 21, 22].

The endoscopic appearance of gastrointestinal metastases is variable. Lesions can be discrete, such as erosions, ulcerations, or nodules, and can extend to gastric linitis with diffuse infiltration [7]. Deep biopsies of the gastric mucosa are recommended because secondary metastases can sometimes only reach the submucosa or seromuscular layer. Superficial biopsies may be negative in 46%–50% of cases [13, 16, 23]. Differential diagnosis from primary digestive lesions is necessary but not always easy. Immunohistochemical staining should be performed to check for estrogen and progesterone receptor expression, which are usually negative in primary gastro‐colorectal tumors. However, 20%–28% of primary gastric carcinomas can express estrogen receptors [24]. TRPS1 and GATA3 immunohistochemical stains should be considered for differentiating primary from secondary breast cancer, while CDX2 is in favor of primary gastric cancer. Survival rates reported in the literature after the diagnosis of gastrointestinal metastases from primary breast cancers vary between 2 and 9 years [5, 25].

## 6 Conclusion

Overall, gastrointestinal metastases from lobular breast carcinomas remain rare. In this context, any new digestive lesion visualized by FDG PET/CT in such patients justifies histological proof, even several years after initial treatment. The appearance of hypermetabolism, especially when it is focal, or changes in the appearance of digestive tract FDG distribution in patients monitored by FDG PET/CT, often attributed to inflammatory pathologies, particularly in patients with associated benign digestive pathologies, should prompt endoscopic explorations and sampling with targeted immunohistochemical analyses in the context of lobular breast carcinoma. From her perspective, the patient understood the value of her follow‐up with FDG PET/CT, recognizing it as a tool that highlighted her recurrence and emphasized the need for histological confirmation in the context of her long‐standing benign gastric inflammatory condition, ultimately leading to a change in therapeutic approach.

## Author Contributions

**Alina Diana Ilonca:** investigation, writing – original draft. **Amélie Gudin‐de‐Vallerin:** investigation. **Sophie Guillemard:** investigation, validation. **Marie‐Claude Eberle:** investigation, validation. **Jeanne Briant:** investigation. **Séverine Guiu:** investigation. **Emmanuel Deshayes:** investigation, visualization. **Cyril Fersing:** visualization, writing – review and editing.

## Consent

Written informed consent was obtained from the individual for the publication of any potentially identifiable images or data included in this article.

## Conflicts of Interest

The authors declare no conflicts of interest.

## Data Availability

Data available on request due to privacy/ethical restrictions.
